# The role of deep hypothermic circulatory arrest in surgery for renal or adrenal tumor with vena cava thrombus: a single-institution experience

**DOI:** 10.1186/s13019-018-0772-z

**Published:** 2018-07-05

**Authors:** Peng Zhu, Songlin Du, Shijun Chen, Shaobin Zheng, Yu Hu, Li Liu, Shaoyi Zheng

**Affiliations:** 1 Department of Cardiovascular Surgery, NanFang hospital, Southern Medical University, 1838 North Guangzhou Avenue, Guangzhou, 510515 People’s Republic of China; 2 Department of Urinary Surgery, NanFang hospital, Southern Medical University, GuangZhou, People’s Republic of China

**Keywords:** Hypothermic arrest, Renal tumor, Thrombectomy, Cardiopulmonary bypass

## Abstract

**Background:**

The aim of this study was to review our experience in managing renal or adrenal tumors with level III or IV inferior vena cava thrombus by using deep hypothermic circulatory arrest (DHCA), and to evaluate survival outcomes.

**Methods:**

Between September 2004 and March 2016, we treated 33 patients with renal or adrenal malignancy tumor and thrombus extending into the inferior vena cava. Patients were identified according to radiographic records and operative findings. Clinicopathological and operative characteristics were recorded, and comparisons of clinical and operative characteristics through DHCA were performed. A Cox regression model was used to determine predictors of perioperative mortality.

**Results:**

Twenty-one out of 33 patients with level III (*n* = 15), level IV (*n* = 5), or level II (*n* = 1) renal or adrenal tumors were treated surgically through cardiopulmonary bypass (CPB) with DHCA, and 12 patients with level II or III tumors were treated surgically through normothermic CPB. Three complications were observed, and one death occurred perioperatively, owing to multiple organ failure. The overall perioperative mortality was 4.7%. There were significant differences in the clinicopathological characteristics, operative duration, estimated blood loss, transfusions and hospital stay depending on use of DHCA. Multivariate analysis indicated that the operative duration (OR, 3.78; *P* < 0.001), estimated blood loss (OR, 1.08; *P* = 0.02), and transfusion (OR, 2.13; *P* = 0.038) during/after surgery were positively associated with higher mortality and morbidity. DHCA failed to reach statistical significance (*P* = 0.378).

**Conclusions:**

Use of CPB and DHCA to treat renal or adrenal tumors allows for complete tumor resection, especially at the T4 stage. Although it can cause physical damage, this technique does not increase operative risk and is a relatively safe approach.

## Background

Tumor thrombus in the inferior vena cava (IVC) occurs in 4–10% of patients with renal cell carcinoma (RCC) and poses a challenge for surgical teams [1]. Because there is no systemic therapy available to significantly decrease tumor burden, surgical intervention is the standard treatment [2]. However, the surgical approach is associated with substantial morbidity and mortality. To minimize operative complications, ensuring adequate exposure and a virtually bloodless surgical field in the upper abdomen and retroperitoneum is essential.

The IVC must be opened when it is associated with tumors. Surgeons therefore must preoperatively determine the precise extent of the tumor and thrombus, to plan their approach accordingly [3]. The use of cardiopulmonary bypass (CPB) with accompanying deep hypothermic circulatory arrest (DHCA) is a recommended and established adjunct technique for surgical management of patients with renal or adrenal tumors and large IVC tumor thrombi [4]. This approach requires close collaboration between urologic and cardiac surgical teams. In our center, the technique of CPB with DHCA is selected by patients undergoing removal of non-metastatic RCC with intrahepatic or subhepatic IVC thrombus. Furthermore, this approach has been used for selected patients with other types of retroperitoneal malignancy with a large IVC thrombus.

Here, we describe our experience with CPB and DHCA in the management of 33 patients with retroperitoneal tumors and large caval thrombi. The aim of this retrospective study was to elucidate the roles of extracorporeal circulation and deep hypothermic total circulatory arrest in surgical treatment for tumor thrombi extending into the retrohepatic caval vein or right atrium. The investigation was performed with special reference to surgical complications, primary mortality and long-term survival.

## Methods

**Fig. 1:**
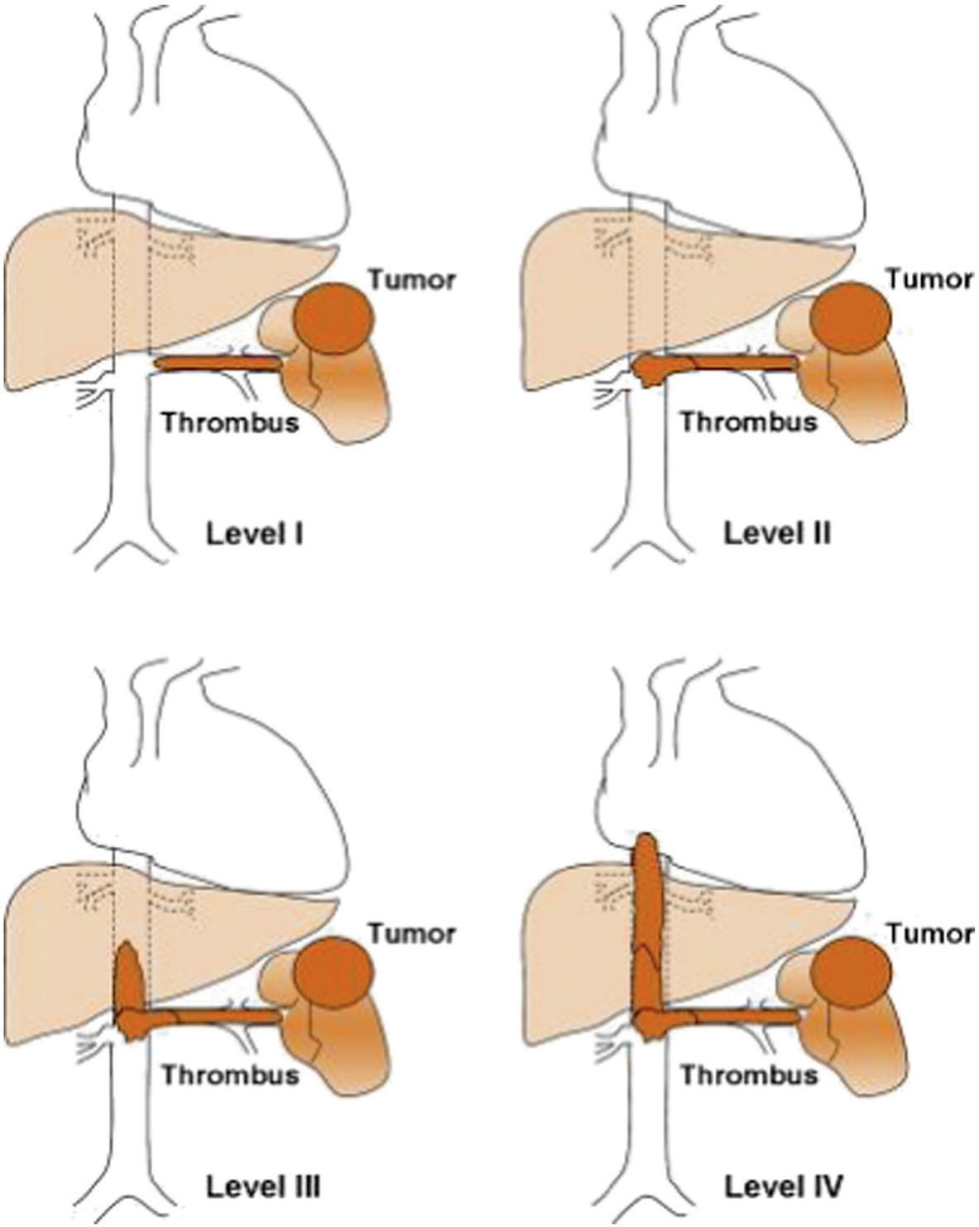
The Mayo classification of macroscopic venous invasion in RCC. Level I: The tumor thrombus is either at the entry of the renal vein or within the IVC < 2 cm from the confluence of the renal vein and the IVC. Level II: The thrombus extends within the IVC > 2 cm above the confluence of the renal vein and the IVC but still remains below the hepatic veins. Level III: The thrombus involves the intrahepatic IVC. The size of the thrombus ranges from a narrow tail that extends into the IVC to one that fills the lumen and enlarges the IVC. Level IV: The thrombus extends above the diaphragm or into the right atrium

From September 2004 to March 2016, 33 patients with large vena caval tumor thrombi from renal or adrenal malignancy underwent surgical resection and removal of thrombi in our center. Among those, 21 patients were treated with the CPB and DHCA techniques. After institutional review board approval was obtained, the demographics, operative data, and postoperative outcomes of these patients were collected retrospectively from our computerized patient database. All surgical procedures were performed by the urologic surgery team in conjunction with the cardiovascular surgery team. Tumor staging was identified on the basis of radiographic records and/or operative findings, and IVC tumor thrombi were classified according to the Mayo Classification of macroscopic venous invasion in RCC (Fig. 1) [5]. Patient and pathological characteristics including age, sex, T classification (T2, T3, or T4), Eastern Cooperative Oncology Group Performance Status (ECOG PS; 0.1, > 1), tumor size, metastatic status (M0 or M1) estimated blood loss (EBL), intraoperative blood transfusions, and perioperative mortality were recorded. The use of DHCA and the length of stay in the intensive care unit were recorded. Patient demographics and perioperative courses are shown in Tables 1 and 2, respectively.

**Table 1:** The patients clinical, pathological and operative characteristics

	DHCA		*P*
	No	Yes	
Number of patients	12	21	
Mean(SD)age,years	54.41(15.43)	47.05(13.86)	0.62
Sex M/F,*n*(%)	6(50%)/6(50%)	14(67%)/7(33%)	0.73
Mean (SD) BMI(kg/m^2^)	22.7(4.4)	22.5(4.8)	0.87
T stage,*n*(%)			0.00
T2	9(75%)	1(5%)	
T3	3(25%)	15(71%)	
T4	0	5(24%)	
Mean(SD)tumour size,mm	8.9(3.4)	9.5(4.2)	0.72
Metastases,*n*(%)			0.35
No	9(75%)	17(81%)	
Yes	3(25%)	4(19%)	
Fuhrman grade,*n*(%)			0.63
2	1(8%)	3(14%)	
3	8(67%)	13(61%)	
4	3(25%)	5(24%)	
ECOG PS,*n*(%)			0.21
0	5(42%)	14(67%)	
1	6(50%)	5(24%)	
> 1	1(8%)	2(10%)	
Mean (SD) Serum creatinine,umol/l	76.37(15.26)	77.12(12.54)	0.62

*M* male, *F* female

**Table 2:** The operative characteristics of patients compared by use of DHCA

Variable	DHCA		P
	No	Yes	
Operative duration,min			0.00
*n*	12	21	
Mean(median)	267.9(96.7)	521.9(120.6)	
EBL,ml			0.01
*n*	12	21	
Mean(median)	854.3(863.8)	5933.7(8040)	
Transfusions,*n*			
*n*	5	21	
Mean(median)	4.8(3.1)	17.22(11.67)	
ICU stay,days			
*n*	1	21	
Mean(median)	6	6.4(1.3)	
Hospital stay,days			0.00
*n*	12	33	
Mean(median)	13.8(4.4)	28.5(14.3)	
Perioperative mortality,*n*			0.58
*n*	12	21	
*N*(%)deaths	0	1(4.7)	
Tumor pathology			
renal clear cell carcinoma	5(41.7)	8(38.1)	
renal cell carcinoma	3(0.25)	5(23.8)	
adrenocortical	2(16.7)	4(19.0)	
Whilms tumor	2(16.7)	3(14.3)	
renal pelvic transitional carcinoma	0	1(4.7)	

**Fig. 2:**
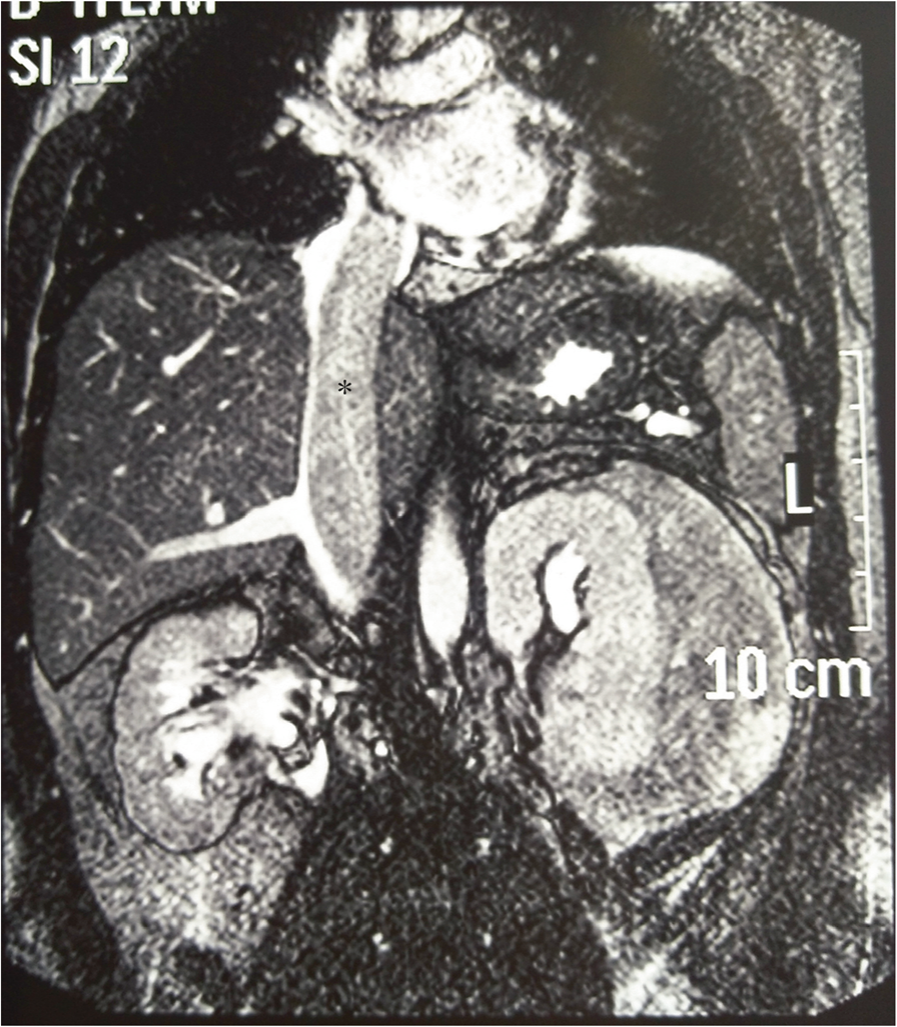
MRI of a left-sided RCC with right atrial tumor thrombus (*)

The preoperative evaluation included abdominal magnetic resonance imaging (MRI) and/or computed tomography (CT) scan, and renal arteriography and/or inferior vena cavography (Fig. 2). Evaluation of the proximal extent of the thrombus is essential for planning appropriate surgical strategies. Right heart catheterization with injection of the superior aspect of the IVC was performed if there was IVC occlusion or if MRI did not clearly demonstrate the distal extent of the thrombus. In current standard practice, when MRI or CT findings are not definitive, transesophageal echocardiography also has reasonable accuracy in assessing the presence and extent of tumor thrombi. X-ray, brain and lung CTs, as well as Positron Emission Tomography-Computed Tomography (PET-CT), were used to evaluated metastases. Renal arterial embolization was performed 48–72 h before surgery in three patients with evidence of a hypervascular IVC tumor thrombus in renal arteriography.

Seven patients with renal or adrenal tumors and large IVC tumor thrombi showed evidence of distant metastatic disease before surgery. A patient with renal clear cell carcinoma was known to have direct tumor extension into the liver.

The hospital charts of all patients were reviewed, and follow-up information was obtained through direct contact with either the patients or local physicians. Complete follow-up data were available for all patients. The follow-up interval ranged from 6 to 60 months, with a mean of 22 months.

### Surgical technique

Radical nephrectomy: Urologists usually choose ventral midline incision, after entering the posterior peritoneal cavity, carefully separating the posterior peritoneal and conglutinated kidney. The IVC showed marked dilation, and tumor thrombi in renal veins or IVC like cords were palpable at that time. Through use of the renal vein as a guide, the renal artery can be accessed and ligated. Separation and perfect traction are important in free renal veins, to allow for ureteral ligation. After opening the sheath of the IVC and the abdominal aorta from the renal hilum, the IVC and renal vein were totally dissociated.

**Fig. 3:**
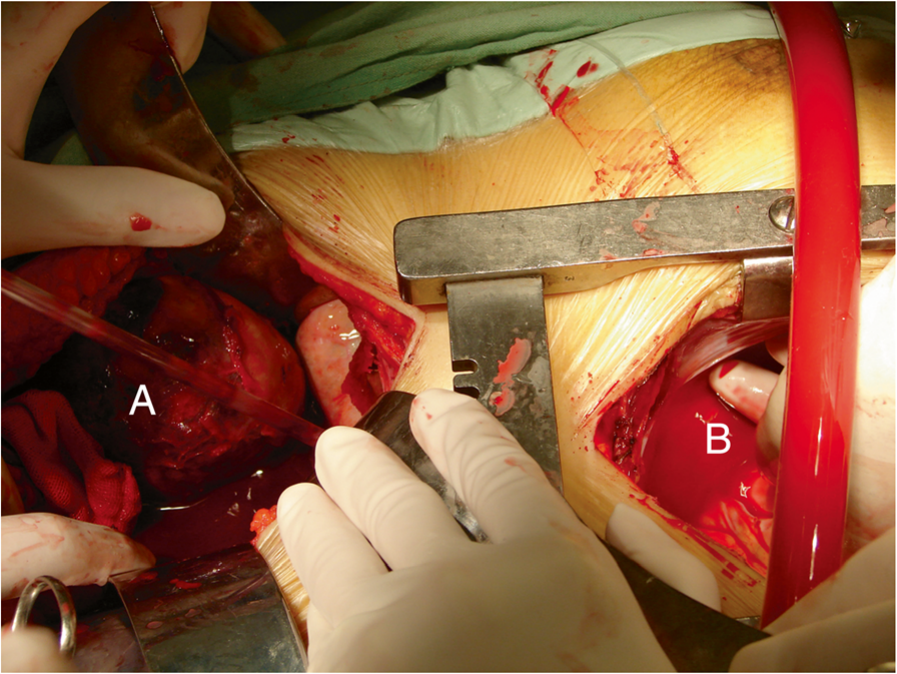
Incisions used for a right-sided tumor. **a** An abdominal rectus incision was made to expose the right renal and IVC. **b** CPB and DHCA were performed through a chest median incision

DHCA and removal of tumor thrombi: The anesthetic considerations for CPB with DHCA have been described in detail elsewhere. The surgical technique was performed according to previously described methods [6]. Briefly, a lateral subcostal abdominal incision was made, and the retroperitoneal tumor mass was mobilized. The entire kidney was mobilized and left attached by only the main renal vein to the tumor thrombus. After maximum mobilization of the tumor, a median sternotomy was performed (Fig. 3). The patient was systemically heparinized. The ascending aorta and the right atrium were cannulated. CPB was performed with systemic cooling, using a topical cold solution in the abdomen and chest. After a core temperature of 18 to 20 °C had been achieved, CPB was terminated, and the blood volume was drained into a pump. Before the onset of circulatory arrest, methylprednisolone (1 g) was administered to protect the brain and vital organs during arrest. The ascending aorta was immediately cross-clamped before circulatory arrest.

**Fig. 4:**
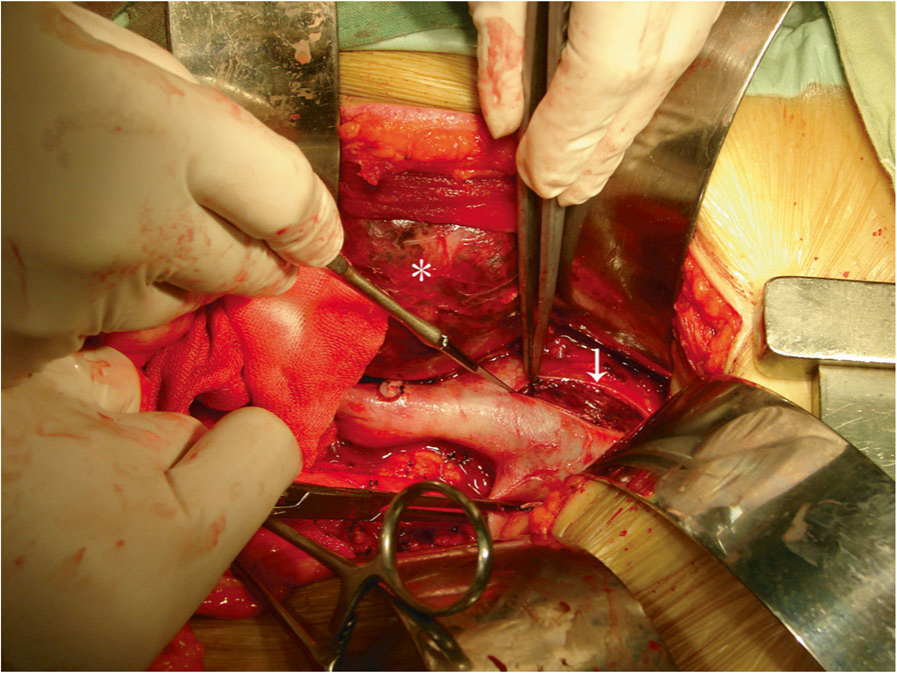
Without blood, the thrombus (arrow) of the hepatic segment was excised and plastic IVC was performed. The right-sided RCC(*)

**Fig. 5:**
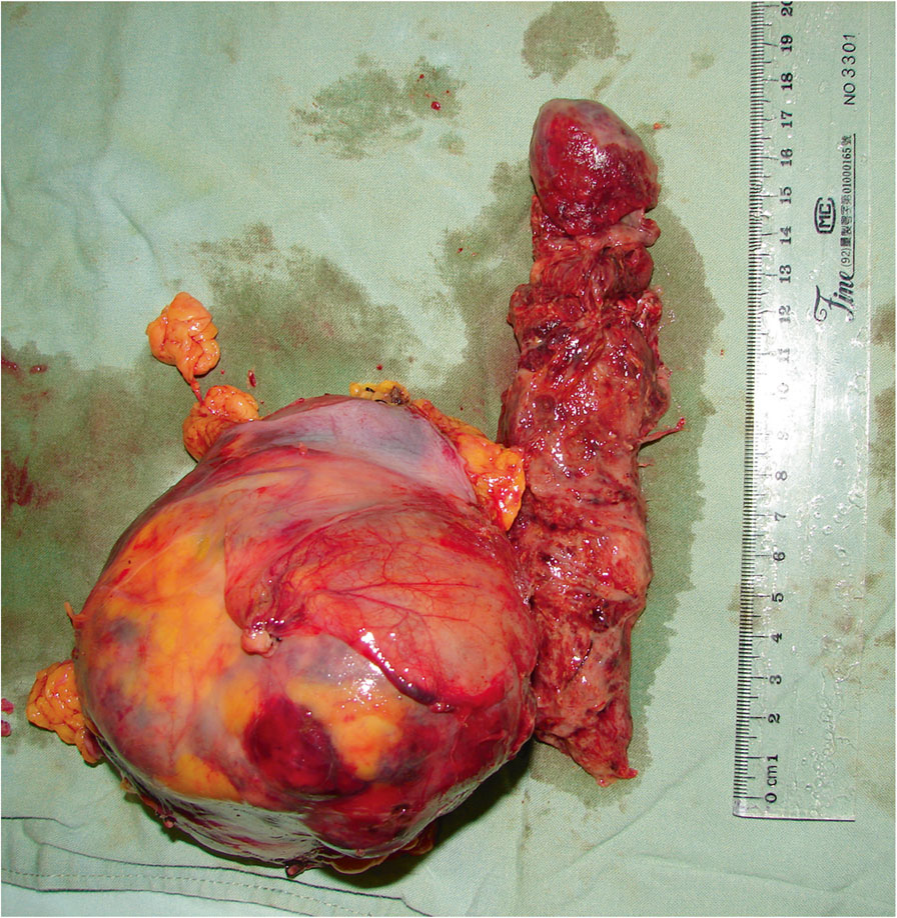
A right-sided RCC with right atrial tumor thrombus invasion

The IVC was mobilized as close to the diaphragm as possible, the RA was opened near the orifice of the IVC, and attention was then directed to the IVC, which was entered near the orifice of the renal vein. The vena caval tumor thrombus was then removed in a bloodless operative field (Fig. 4). After extraction of the thrombus and repair of the caval wall, a two-stage venous cannula was reinserted into the RA, and the right atrium was closed. The CPB was reinstituted, and the patient was rewarmed to a core temperature of 37 °C. During the rewarming procedure, radical nephrectomy was also performed. CPB was then terminated, and heparin was reversed. In all cases, the primary malignancy and IVC tumor thrombus were completely removed (Fig. 5). Atriotomy was performed in five patients with tumor thrombi extending into the right atrium and in selected other patients with friable or adherent thrombi.

Two patients underwent vena caval reconstruction with pericardial grafts. Additional procedures done at the time of CPB with DHCA included coronary artery bypass grafting in one patient with renal clear cell carcinoma, pulmonary resection of metastases in one patient with RCC and hepatic lobectomy in the patient with invasive adrenocortical carcinoma.

Comparisons of patient demographics and operative characteristics were performed between bypass techniques. For the risk analysis, a multiple logistic regression model was developed by using a forward stepwise variable selection method. Fisher’s exact test or chi-square test was used for categorical variables, and Student’s *t*-test was used for continuous covariates. *P* values < 0.05 were considered statistically significant. Analyses were performed in SPSS version 20.0 (IBM Corporation, Somers, NY, USA).

## Results

Between September 2004 and March 2016, 33 consecutive patients (20 males, 13 females) undergoing surgery were identified. The clinicopathological characteristics of all patients are summarized in Table 1. At the time of surgery, the median age was 50 years (IQR: 18 to 72 years). The tumor was located on the left in six (18.2%) patients and on the right in 27 (81.8%) patients.

The renal tumors had a median size of 9.3 (IQR: 6–15 cm). The median total operation time was 521 min, with median CPB and DHCA durations of 191 min and 40.5 min, respectively, and the median total operation time was 267 min without CPB.

In the CPB with DHCA group, the median estimated blood loss was 5933 ml (IQR: 1000 to 26,000), and all patients required blood transfusions, with a median estimated RBC transfusion volume of 17.22 U (IQR: 7–45).At the beginning of the study, three patients had uncontrollable hemorrhage during surgical removal of the tumor,and an extracorporeal circulation with DHCA techniques were urgently established to complete the surgery. The blood loss and transfusion volume in the three patients caused statistical fluctuations.The decision to administer blood was based on preoperative hemoglobin levels and the degree of blood loss. The overall perioperative mortality was 4.7%.

Only one operative death occurred, in a 51-year-old woman with severe congestive cardiac failure. This patient experienced intraoperative heart failure and could not be weaned from CPB. Three complications were observed in this patient cohort: re-operation because of bleeding (*n* = 1), acute renal failure (n = 1) and severe post-operative subcutaneous hydrops. All of the remaining complications were resolved with appropriate treatment. There were no other ischemic or neurologic complications. There were no cases of perioperative tumor embolization. The average hospital stay of surviving patients was 20 days (range: 8–56 days), and 6.4 days in the intensive care unit (range: 4 to 8 days). Pathologic examination of the tumors revealed that 13 (39.3%) patients had renal clear cell carcinoma, eight (24.2%) patients had RCC, six (18.1%) patients had adrenocortical carcinoma, five (15.1%) patients had Wilms’ tumor, and one (3%) patient had renal pelvic transitional carcinoma.

Comparisons of patient characteristics based on the bypass technique are shown in Table 2. There were no differences in patient perioperative mortality between patients undergoing either bypass technique. The pathological (T stage), operative duration, EBL, transfusion, and hospital stay differed depending on bypass techniques,and tumor pathology were listed in Table 2.

Risk factors potentially contributing to perioperative mortality were assessed. Multivariate analysis indicated that operative duration (OR, 3.78; *P* < 0.001), estimated blood loss (OR, 1.08; *P* = 0.02), and transfusion (OR, 2.13; *P* = 0.038) during/after surgery were each positively associated with increased mortality and morbidity. DHCA failed to reach statistical significance (*P* = 0.378).

The median follow-up time for the patients was 24 months (range: 6–60 months) after surgery. The mean overall survival period of the cohort was 34.5 ± 6.7 months, and the disease-free survival period was 26.6 ± 17.4 months. After surgery, liver metastases were found in five patients, and lung metastases were found in four patients. Abdominal metastases were found in two patients at 3 months after surgery: one had been alive for 22 months, and the other had been alive for 7 months. The opposite renal metastases were found in two patients at 4 and 36 months after surgery, who had been alive for 6 months and 48 months. Seven patients with metastases refused further treatment, and follow-up was lost; the other patients with metastases were administered chemotherapy and/or targeted therapy. The mean overall survival of patients without metastases was 38.3 ± 22.4 months.

## Discussion

Among retroperitoneal tumors, including renal and retroperitoneal sarcoma, renal pelvic transitional cell carcinoma, Wilms’ tumor pheochromocytoma, adrenocortical carcinoma and some lymphomas, RCC is most often found to invade the IVC, even in the absence of distant metastases [7]. RCC has a biological propensity toward intravascular invasion of the renal vein and extension into the IVC. Neoplastic invasion of the IVC occurs in 5–15% of patients with renal cancer, and the tumor embolus extends to the right atrium in 14–39% of patients [8]. Renal or adrenal tumor with IVC tumor thrombus often implies an unfavorable prognosis that is unsuitable for surgery. Radical nephrectomy together with thrombectomy is the most effective therapeutic method after medical therapies have proven ineffective [9]. However, surgical management for patients with RCC with a tumor thrombus extending into the IVC, or even the right atrium, poses a major surgical challenge. Although renal tumors often extend into the IVC before clinical diagnosis, unlike other tumors, their prognosis is fairly good if the tumor is surgically resected and metastatic disease is absent [10].

Several surgical techniques for the treatment of these tumors have been proposed, but they are limited by small patient numbers and limited follow-up [1–3]. Additionally, several reports have combined patients with different bypass techniques and different levels of thrombi. Moreover, substantial controversy regarding the best operative management method still persists regarding treatment of RCC patients with level III or IV thrombi. Some surgeons have reported the use of CPB without circulatory arrest and vascular occlusion without CPB to facilitate the removal of tumor thrombi. Level IV thrombi can be managed with veno-venous bypass and/or caval-atrial shunt [11, 12]. Although high-level thrombi can be removed successfully without CPB and DHCA, kidney health and liver function may be affected postoperatively, even when the procedures are performed by experienced surgeons. In our institution, our experience suggests that use of CPB and DHCA decreases the risk of life-threatening intra-operative hemorrhage and incomplete thrombus removal. As performed by a urological surgery team in conjunction with a cardiovascular surgery team, CPB and DHCA have been safely used to treat RCC with high-level IVC thrombi.

The use of DHCA has been questioned in surgical resection, because there is concern regarding the risk of hemorrhage with heparinization and neurological sequelae with low-flow cerebral perfusion. CPB with hypothermia may be associated with platelet dysfunction. However, when CPB is combined with systemic use of heparin, coagulopathy and bleeding from the retroperitoneum are risks. The present study and our data demonstrated CPB with DHCA to be safe in cases of level III or IV tumor thrombus. The deep hypothermia in conjunction with circulatory arrest, thought to limit renal and liver ischemia in thrombus removal, may protect the contralateral kidney and decrease postoperative dialysis. Hence, CPB with DHCA may potentially decrease perioperative mortality. Hatcher et al. [13] have reported that patients who underwent extraction of a mobile tumor thrombus from the vena cava had a 69% 5-year survival (median, 9.9 years), whereas patients with tumor thrombi directly invading the vena cava had a 26% 5-year survival (median, 1.2 years), which improved to 57% (median 5.3 years) when the involved vena caval side wall was resected successfully. In such cases, curative resection might be feasible with reasonable long-term survival.

Surgical techniques have been developed to remove tumor thrombi extending into the retrohepatic caval vein and the right atrium [14] These procedures are accompanied by risks of unexpected and life-threatening hemorrhage with hypotension, disruption, and embolization of the tumor into the pulmonary arteries, myocardial infarction, stroke, renal failure, and hepatic dysfunction [15]. In contrast, the use of DHCA can decrease the risks of warm hepatic and renal ischemia and of incomplete tumor extirpation. This technique allows adequate hemodynamics to be maintained during long surgical interventions in several serous cavities. Moreover, it allows for removal of thrombi and tumorous cells from cardiac cavities, and prevention of hematogenous debrimetatases from intravascular tumors. The necessity for DHCA is debatable if the IVC can be completed within 10 min in every case [16]. However, longer times are occasionally needed for meticulous curetting or complicated reconstruction. In addition, because the situation cannot be predicted in advance, we use deep hypothermia to minimize neurologic complications.

Akchurin et al. [17] have demonstrated that CPB and the cell-saver technique in combination with oncologic and cardiovascular surgery does not increase the risk of hematogenous dissemination. They studied eight patients who underwent oncologic surgery and open heart surgery or procedures on major blood vessels. In three patients, the intravascular portion of the tumor was extracted as much as possible through a right atrium approach (in three cases the tumor invaded the IVC). All of the patients had uneventful postoperative periods and were alive 1 year after the procedures. During cytological investigation after each operation, tumor cells were found only on the internal surfaces of the heart-lung machine arterial filters with 20 μm holes. That study suggests that special cardiovascular devices such as the heart-lung machine and cell saver might be used in borderline situations in oncology without increasing the risk of hematogenous tumor dissemination. In our hospital, we suspended blood reclamation when the whole tumor embolus was exposed. After the tumor thrombus was carefully dissected from the IVC wall and removed en bloc, we rinsed the abdominal cavity with saline repeatedly, then resumed blood reclamation. This procedure enables surgeons to re-infuse lost blood and avoid transfusion with donated blood to overcome immune and post transfusion complications. Furthermore, the current approach decreased the risk of hematogenous dissemination resulting from aspiration of the tumor cells from the surgical wound.

## Limitations

Our study has several limitations. A limited number of patients were enrolled, and the follow-up time was relatively short. We used CPB with DHCA alone in surgical resection without other techniques. A randomized controlled trial should be conducted in the future to compare this technique with other surgical techniques for the management of RCC with level III or IV tumor thrombus. Finally, we were unable to perform adjustment for surgical-team or surgeon-specific characteristics that might have made the analysis more robust.

## Conclusions

Renal carcinoma with tumor extension into the IVC presents a major surgical challenge. Surgery for RCC and high-level tumor thrombus by using CPB with DHCA, a preferred surgical technique, provides better visibility of the vascular anatomy, thus allowing for complete extraction of the thrombus, regardless of its invasiveness and potential for adverse effects. In addition, this technique significantly decreases both the rate of severe intraoperative complications and the perioperative mortality. These results suggest that long-term survival is possible if radical nephrectomy and complete extirpation of the tumor thrombus are performed, and the use of CPB with DHCA and retrograde cerebral perfusion results in favorable outcomes. Further prospective studies are needed to evaluate the possible benefits of DHCA and other surgical techniques to decrease the potential risk of cavoatrial tumor extension.

## Abbreviations

CPB: Cardiopulmonary bypass
DHCA: Deep hypothermic circulatory arrest
EBL: Estimated blood loss
IBS: Intraoperative blood salvage
IVC: Inferior vena cava
MRI: Magnetic resonance imaging
OR: Odds Ratic
PET-CT: Positron emission tomography-computed tomography
RCC: Renal cell carcinoma
VVBP: Venovenous bypass
